# Retroperitoneal unicentric Castleman's disease (giant lymph node hyperplasia): case report

**DOI:** 10.1590/S1516-31802007000400013

**Published:** 2007-07-01

**Authors:** Jaques Waisberg, Marie Satake, Nagamassa Yamagushi, Leandro Luongo de Matos, Daniel Reis Waisberg, Ricardo Artigiani Neto, Maria Isete Fares Franco

**Keywords:** Castleman disease, Retroperitoneal neoplasms, Lymphatic system, Lymphoproliferative disorders, Retroperitoneal space, Doença de Castleman, Neoplasias retroperitoneais, Sistema linfático, Transtornos linfoproliferativos, Espaço retroperitoneal

## Abstract

**CONTEXT AND OBJECTIVE:**

Castleman's disease, or giant lymph node hyperplasia, is a rare disorder of the lymphoid tissue that causes lymph node enlargement. It is considered benign in its localized form, but aggressive in the multicentric type. The definitive diagnosis is based on postoperative pathological findings. The aim here was to describe a case of retroperitoneal unicentric Castleman's disease in the retroperitoneum.

**CASE REPORT:**

A 61-year-old white male with weight loss and listlessness presented with moderate arterial hypertension and leukopenia. Abdominal tomography revealed a 5 × 4 × 5 cm oval mass of low attenuation, with inner calcification and intense enhancement on intravenous contrast, located in the retroperitoneal region, between the left kidney and the aorta, at the renal hilus. Exploratory laparotomy revealed a non-pulsatile solid oval mass situated in the retroperitoneum, adjacent to the left renal hilus. The retroperitoneal lesion was removed in its entirety. Examination of frozen samples revealed benign lymph node tissue and histopathological examination of the surgical sample revealed hyaline-vascular giant lymph node hyperplasia (Castleman's disease). The patient was discharged on the 12^th^ day without significant events. Two months after the operation, the patient was readmitted with severe cardiac insufficiency, acute renal failure and bronchopneumonia, which progressed to acute respiratory insufficiency, sepsis and death.

## RESUMO

**CONTEXTO E OBJETIVO:**

A doença de Castleman ou hiperplasia linfonodal gigante é desordem rara do tecido linfóide que causa linfadenomegalia. É considerada afecção benigna em sua forma localizada, porém agressiva no tipo multicêntrico. O diagnóstico definitivo está baseado nas achados anatomopatológicos pós-operatórios. O objetivo foi descrever um caso de doença de Castleman (hiperplasia linfonodal gigante) unicêntrica retroperitoneal localizada no retroperitônio.

**RELATO DO CASO:**

Homem, 61 anos, branco, com emagrecimento e adinamia, apresentava hipertensão arterial moderada e leucopenia. A tomografia abdominal revelou massa ovalada de 5 × 4 × 5 cm, hipoatenuante, com calcificação de permeio e intenso realce ao contraste intravenoso, localizado na região retroperitoneal, entre o rim esquerdo e a aorta, no hilo renal. A laparotomia exploradora revelou massa sólida, não pulsátil, ovalada, situada no retroperitônio, próximo ao hilo renal esquerdo e que foi retirada completamente. O exame de congelação mostrou tecido linfonodal benigno e o exame histopatológico da peça cirúrgica revelou hiperplasia linfonodal gigante (doença de Castleman), forma hialina-vascular. O doente obteve alta no 12o dia. Após dois meses da operação, reinternou com quadro de insuficiência cardíaca grave, insuficiência renal e broncopneumonia, evoluindo com insuficiência respiratória aguda, sepse e óbito.

## INTRODUCTION

Castleman's disease (CD) is a rare lymphoproliferative disorder characterized by hyperplasia of lymphoid follicles, with proliferation of mature lymphocytes and/or plasma cells.^1^ Also known as angiofollicular giant lymph node hyperplasia, the clinical manifestations of CD are heterogeneous, ranging from asymptomatic discrete lymphadenopathy to recurrent episodes of diffuse lymphadenopathy with severe systemic symptoms. Over the last decade, a large body of evidence has given support to the view that infection with human herpesvirus 8 (HHV-8 or Kaposi's sarcoma-associated herpesvirus) is important in the etiology and management of CD.^2^ In this paper we present a patient with abdominal Castleman's disease, with retroperitoneal location. This rare disease should therefore be added to the long list of diseases in the differential diagnosis of abdominal masses.^3^

## CASE REPORT

A 61-year-old white man was hospitalized with a six-month history of weight loss, listlessness and exertional dyspnea. He had rheumatic fever and had had mitral and aortic insufficiency since the age of 18 years, which had more recently been treated with captopril, furosemide, digoxin and spironolactone. The patient had had Hyde's nodular prurigo since the age of 24 years, which had been treated with thalidomide over the last 3.5 years. He presented a picture of severe depressive disorders and dementia, with memory impairment, together with behavioral changes and suicidal tendencies for which he was receiving chlorpromazine.

Physical examination revealed only moderate arterial hypertension without superficial lymphadenomegaly, while laboratory tests showed leukopenia (2970/mm^3^) and discrete anemia (hemoglobin 11.6 g/dl). The values for C-reactive protein, alpha-1 acid glycoprotein and streptococcal antibody titer, along with the erythrocyte sedimentation rate, were all within normal limits. The level of lactate dehydrogenate was mildly elevated. Chest radiography did not reveal any mediastinal masses. The leukopenia and anemia were attributed to the use of drugs with myelotoxic potential (chlor­promazine, spironolactone and captopril) and folate metabolism inhibition associated with inadequate food intake. Following the suspension of these drugs and administration of folinic and folic acids, the leukocyte count rose to 6440/mm^3^ and the hemoglobin rate normalized. Abdominal and pelvic computed tomography (CT) demonstrated an oval mass measuring 5 × 4 × 5 cm of low attenuation, with inner calcification and intense enhancement on intravenous contrast, located in the retroperitoneal region, between the left kidney and the aorta, near the renal hilus. CT of the neck and chest did not reveal any abnormalities. Renal and hepatic functions were normal, while anti-HIV and anti-HTLV-1 and 2 (anti-human T-cell lymphotrophic virus types 1 and 2) tests were negative. A myelogram showed no abnormalities. The concentrations of IgG anti-toxoplasmosis antibody and IgG anti-cytomegalovirus antibody were clinically insignificant. Serum protein electrophoresis demonstrated absence of monoclonal or polyclonal hypergammaglobulinemia.

The patient underwent exploratory laparotomy that revealed a non-pulsatile solid oval mass. This was situated in the retroperitoneum, adjacent to the left renal hilus, and did not involve the renal vessels. The liver and spleen were seen to be macroscopically conserved. The remainder of the abdominal cavity presented no abnormalities, and no other masses or enlarged lymph nodes were observed. The retroperitoneal lesion was removed in its entirety, with all elements of the left renal hilus remaining intact. There was no evidence of invasion into the adjacent tissues.

**Figure 1.**
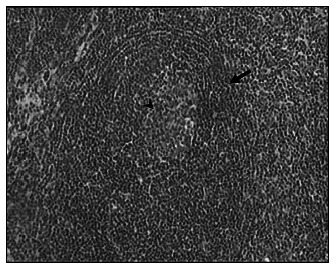
Photomicrograph (hematoxylin and eosin, 40 X) of hyaline-vascular Castleman's disease. Note the concentric rings (arrows) of small lymphocytes surrounding an atrophic follicle and a hyalinized vessel (arrowhead) entering the follicle.

**Figure 2.**
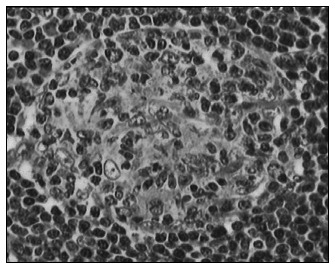
Photomicrograph (hematoxylin and eosin, 100 X) showing a follicle with small germinal center and interfollicular capillary proliferation. Marked proliferation of small blood vessels with endothelium, some of it hyalinized.

Examination of frozen samples revealed benign lymph node tissue. Histopathological examination of the surgical sample revealed hyaline-vascular Castleman's disease (giant lymph node hyperplasia) (Figures 1 and 2). The patient was discharged on the 12^th^ day, without significant events.

Over the weeks that followed, the patient's mental condition significantly worsened, with severe depressive episodes, confused state and behavioral disturbances. Two months after the operation, the patient was readmitted with severe cardiac insufficiency, acute renal insufficiency and bronchopneumonia. Chest radiography showed pulmonary congestion and condensation in the base of the left lung. No mediastinal masses were observed. The patient progressed with acute respiratory insufficiency and sepsis, and died two days later. Authorization for autopsy was denied.

## DISCUSSION

The majority of the intra-abdominal lesions in CD are located in the pelvis, mesentery and perinephric regions.^3,4^ CD has two main histological variants: the hyaline vascular type, comprising 80-90% of the cases, and the plasma cell type comprising the remaining 10%-20%.^1,3,5^ Recognition of patients with both localized lymphadenopathy and disseminated disease led to an additional clinical categorization for CD: unicentric (UCD) versus multicentric (MCD). Another histological type, known as plasmablastic MCD has been described in particularly aggressive cases of MCD, in association with POEMS syndrome (polyneuropathy, organomegaly, endocrinopathy, monoclonal proteins and skin changes), HHV-8 infection and progression to plasmablastic lymphoma.^6^

The unicentric hyaline-vascular variant (U-HVV) generally affects young people and occurs in approximately 70% of the patients with CD. Many patients with mediastinal or abdominal lymphadenopathy are either asymptomatic or alerted to their conditions via radiographic examinations or surgical procedures for other conditions,^2,7^ as was the case in our patient, or their conditions come to medical attention because of compressive symptoms. The first case series found that the mediastinum was the most common location for lymph node enlargement, but subsequent series have found that cervical, abdominal and axillary lymphadenopathy are equally common.^8^ U-HVV is characterized by small hyaline-vascular follicles and interfollicular capillary proliferation.^4,5,7^ Because of its vascularization, there is risk of massive hemorrhage during surgery.^5^

The unicentric plasma cell variant (U-PCV) occurs in approximately 20% of patients with CD. It is characterized by hypertrophy of a single lymph node chain. The majority of such patients present with constitutional symptoms. Anemia and elevated sedimentation rate are present in most cases.^2^ Microscopically, the distinguishing features of U-PCV are sheets of mature plasma cells in the interfollicular tissue and a normal to large-sized follicular center.^1,3^

The multicentric plasma cell variant (M-PCV) is the least commonly encountered CD variant (10%), but presents with the most protean manifestations. Patients with M-PCV are typically older than those with unicentric disease and are more likely to have systemic symptoms. The most common of these are anemia, fever, diaphoresis, weight loss, night sweating and fatigue.^1,2,9^ Patients with M-PCV may have an elevated erythrocyte sedimentation rate, hyperglobulinemia, hypoalbuminemia, polyclonal hypergammaglobulinemia, leukocytosis, thrombocytosis or splenomegaly.^1,4^ In contrast to the localized form, the clinical course of the multicentric form may be chronic, with remissions and exacerbations requiring continuous therapy, or it may be malignant, rapidly progressive and fatal.^4,7^ POEMS syndrome, seen in up to 15% of M-PCV cases, is thought to result from plasma-cell dyscrasia and subsequent production of monoclonal proteins.^9^ Patients with M-PCV and HIV-infection are at increased risk of developing non-Hodgkin's lymphoma.^10^ Death in M-PCV cases may be caused by sepsis, systemic inflammation leading to multiorgan systemic failure, or the development of malignancy, most commonly lymphoma.^2^

Although the radiographic findings in cases of the disease are non-specific, hypoechoic, well-defined, localized abdominal masses with enhancement on dynamic CT, probably due to their high vascularization, these may be considered indicators of CD,^1,3,7^ as seen in the patient of the present case report. One distinguishing radiological finding in cases of hyaline vascular CD is the central area of low attenuation, which corresponds to the central stellate fibrosis seen in gross pathological examination.^1,7^ From radiological and clinical points of view, the findings in cases of disseminated CD are indistinguishable from those of lymphoma. Therefore, biopsy of the enlarged lymph node is essential for making the diagnosis.^1^ Immunohistochemical stains for the κ and λ chains confirm the presence of a polyclonal process and, when in association with positive immunostaining for CD20, this suggests CD.^4^

In cases of patients with lymph node histology consistent with CD, other causes of reactive lymphadenopathy that look similar should first be ruled out, such as: rheumatoid arthritis, lupus, Sjögren syndrome, HIV infection, lymphoma and drug sensitivity.^2^

Unicentric CD of either the hyaline vascular or plasma cell variants is almost universally cured after resection of the lymph nodes involved and has not been associated with increased mortality.^2^ Radiotherapy may be a viable option for patients who are poor surgical candidates. Because of the disseminated nature of the lymphadenopathy seen in multicentric disease, complete surgical debulking is rarely possible.^2,8^ The treatment method for the multicentric form has not yet been firmly established, but systemic therapy with steroids or antiblastic agents has been used with variable success.^4^ Ganciclovir, interferon-α or rituximab may be the best treatment options for patients with HHV-8 infections, while cyclophosphamide, vincristine, doxorubicin and either prednisone (CHOP) or dexamethasone (CVAD) may be the most appropriate for patients with severe systemic manifestations of MCD.^2^ Thalidomide has immunomodulatory properties and may act specifically to decrease the production of interleukin-6, which is a potent stimulant for the production of B cells, and thus it may participate in the pathogenesis of CD.^11^As our patient had received thalidomide to treat Hyde's nodular prurigo, we can speculate that this drug may have contributed towards the absence of any symptomatic manifestation of CD.

Appropriate follow-up should be tailored to the CD variant and symptoms. Patients with unicentric disease without systemic involvement should have an additional radiological assessment 6-12 months after therapy, to verify the cure, with additional testing or therapy only pursued in the event of the onset of new symptoms.^2^ For patients with MCD, close follow-up and periodic surveillance are necessary, so that concurrent or ensuing malignant lesions can be detected. The prognosis is poor in the plasma cell type, particularly in multicentric CD, because of the high incidence of malignancy.^1,3,4^

## References

[1] Kim TJ, Han JK, Kim YH, Kim TK, Choi BI (2001). Castleman disease of the abdomen: imaging spectrum and clinicopathologic correlations. J Comput Assist Tomogr.

[2] Casper C (2005). The aetiology and management of Castleman disease at 50 years: translating pathophysiology to patient care. Br J Haematol.

[3] Kimura T, Inoue T, Katayama K, Hirose K, Imamura Y, Yamaguchi A (2002). Mesenteric Castleman's disease: report of a case. Surg Today.

[4] LeVan TA, Clifford S, Staren ED (1989). Castleman's tumor masquerading as a pancreatic neoplasm. Surgery.

[5] Hung IJ, Kuo TT, Lin JN (1992). New observations in a child with angiofollicular lymph node hyperplasia (Castleman's disease) originated from the mesenteric root. Am J Pediatr Hematol Oncol.

[6] Menke DM (2000). Ly-1b cells and Castleman disease. Blood.

[7] Ferreirós J, Gómez León N, Mata MI, Casanova R, Pedrosa CS, Cuevas A (1989). Computed tomography in abdominal Castleman's disease. J Comput Assist Tomogr.

[8] Herrada J, Cabanillas F, Rice L, Manning J, Pugh W (1998). The clinical behavior of localized and multicentric Castleman disease. Ann Intern Med.

[9] Peterson BA, Frizzera G (1993). Multicentric Castleman's disease. Semin Oncol.

[10] Oksenhendler E, Duarte M, Soulier J (1996). Multicentric Castleman's disease in HIV infections: a clinical and pathological study of 20 patients. AIDS.

[11] Franks ME, Macpherson GR, Figg WD (2004). Thalidomide. Lancet.

